# Cytokine and Chemokines Alterations in the Endemic Form of Pemphigus Foliaceus (Fogo Selvagem)

**DOI:** 10.3389/fimmu.2017.00978

**Published:** 2017-08-14

**Authors:** Rodolfo Pessato Timóteo, Marcos Vinicius Silva, Djalma Alexandre Alves da Silva, Jonatas Da Silva Catarino, Fernando Henrique Canhoto Alves, Virmondes Rodrigues Júnior, Ana Maria Roselino, Helioswilton Sales-Campos, Carlo José Freire Oliveira

**Affiliations:** ^1^Laboratory of Immunology, Institute of Natural and Biological Sciences, Federal University of Triângulo Mineiro, Uberaba, Brazil; ^2^Division of Dermatology, Department of Medical Clinics, Ribeirão Preto Medical School, University of São Paulo, Ribeirão Preto, Brazil

**Keywords:** pemphigus foliaceus, fogo selvagem, IL-22, cytokines, chemokines, pathogenesis

## Abstract

**Introduction:**

The endemic form (fogo selvagem—FS) of pemphigus foliaceus is an autoimmune disease characterized by the presence of IgG autoantibodies against desmoglein-1. Despite the array of findings, the role of chemokines and cytokines that dictate the immune response and disease outcome is still poorly investigated.

**Materials and methods:**

Serum from 64 patients diagnosed with FS was used to draw and establish the levels of these molecules on this disease and establish the levels of these molecules with the severity of FS, and influence of treatment.

**Results:**

In comparison to healthy subjects, FS patients, newly diagnosed and still without therapeutic intervention, had higher levels of IL-22 and CXCL-8, and reduced levels of IFN-γ, IL-2, IL-15, and CCL-11. Furthermore, treatment using immunosuppressant drugs augmented the production of IFN-γ, IL-2, CCL-5, and CCL-11 besides reducing the levels of IL-22 and CXCL-10. Immunosuppressive therapy seemed to have long-lasting effects on the production of higher amounts of IFN-γ, IL-2, and CCL-5, besides keeping lowered the levels of IL-22 in remission FS patients.

**Conclusion:**

Taken together, our findings suggest a putative role of IL-22 in the pathogenesis of FS. Finally, data presented here may contribute for better understanding the immune aspects that control disease outcome.

## Introduction

Pemphigus is a severe and rare autoimmune disease characterized by antibodies targeting desmosomal proteins that are crucial to mucosal and epidermal integrity. So far, two major types of pemphigus were described, pemphigus vulgaris and pemphigus foliaceus (PF), and in some cases, these diseases can be subcategorized or present variants (1). PF can be found in all continents, but its endemic form known as “fogo selvagem” (FS) is more frequently found in Brazil, where the disease is up to 20 times more frequent than in other committed countries such as Peru, Colombia, Algeria, and Tunisia (2). Particularly in Brazil, autoimmune blistering diseases, such as PF, are more frequently associated with black subjects living in rural areas (1, 3, 4).

Fogo selvagem shares clinical and immunopathological features to the non-endemic form and is characterized by the presence of pathogenic autoantibodies (primarily IgG4) against the desmoglein-1 (Dsg1), resulting in loss of organization between keratinocytes (acantholysis) that leads to the formation of intraepidermal vesicles (1, 5, 6). Despite the role of antibodies targeting desmoglein, several other aspects are associated with the complex pathogenesis and susceptibility of pemphigus. Increased levels of T helper-2 (Th2) cytokines such as IL-4, IL-10, and IL-13 were already shown to be involved in the production of IgG4 by B-lymphocytes in both PF and PV patients. Those patients also exhibited reduced levels of IL-2 and IFN-γ, resulting in suppressed expansion of Th1 lymphocytes, which suggests an inhibitory effect of Th2-cytokines, thus contributing to disease worsening (7).

Owing to the immune imbalance, pharmacological treatment of pemphigus, especially in underdeveloped and developing countries, is mainly based on immune modulatory and anti-inflammatory drugs such as glucocorticoids (GC). However, some patients are refractory or may present some side effects (8). This poor responsiveness could be related to elevated levels of the inflammatory cytokines IL-6 and TNF-α, which can induce GC resistance observed in peripheral blood mononuclear cells (PBMCs) isolated from pemphigus subjects (8). Furthermore, augmented levels of IL-10 and IL-12 concomitant to reduced levels of IL-2, IL-4, IL-5, and IFN-γ were found in PF patients without treatment, when compared to their control counterparts (9). On the other hand, PBMCs isolated from PF patients treated with GC, showed increased levels of both the inflammatory cytokine IL-1β or in the IL-5/IFN-γ, when compared to their healthy counterparts (10). Taken together, the few studies conducted so far only describe cytokines related to Th1 and Th2 pattern of immune response. In other words, these findings do not permit a clear definition of the entire immune aspects related to the onset, treatment, and remission of the disease.

In the last years, other cytokines such as IL-9, IL-17, and IL-22 have been implicated in the pathogenesis of inflammatory and autoimmune diseases affecting skin, such as psoriasis, for example (11, 12). IL-9 seems to act synergistically with IL-17 and IL-22, which can be related to disease worsening (12). Though skin diseases such as psoriasis can be associated with the development of pemphigus (13, 14), the latter still requires more clarification regarding cytokine interplay, immune profile, and the impact of treatment on disease outcome.

Thus, given the lack of data concerning the immune profile of FS patients in different clinical stages of disease as well as the impact of treatment in this context, this study aimed to elucidate the cytokine/chemokine interplay in those subjects.

## Materials and Methods

### Patients

**Figure 1:**
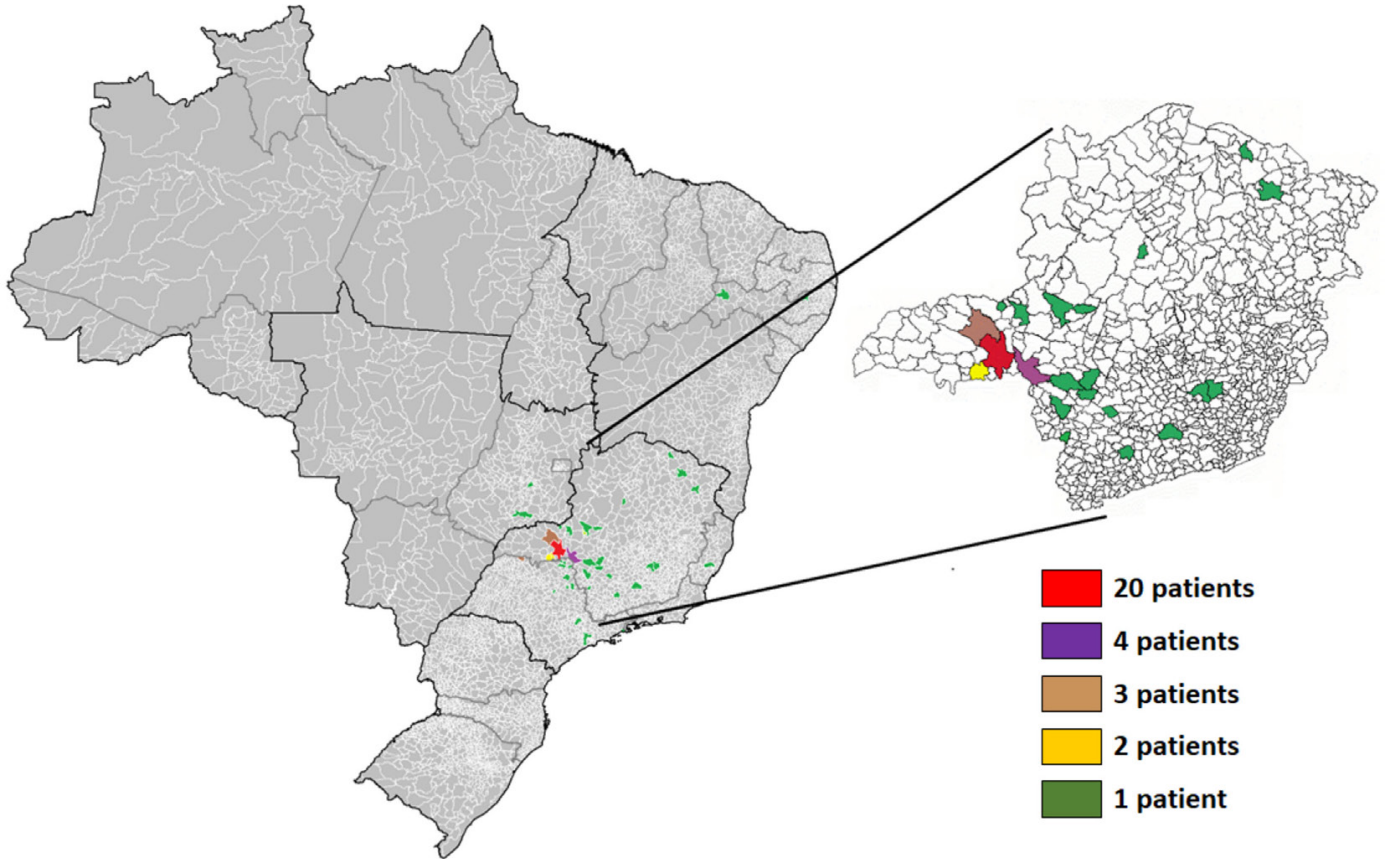
Geographical distribution of patients with the endemic form (fogo selvagem) of pemphigus foliaceus. The respective colors represent the number of patients from each municipality. Highlight to the state of Minas Gerais.

The study was conducted with patients from a hospital for blistering diseases located in Uberaba, state of Minas Gerais, Brazil, from January 2013 to December 2015. Serum from 64 patients diagnosed with FS was used in this study (Table 1; Figure 1). Samples were collected during the consultation of patients in different stages of treatment (before, during, and after treatment) in the hospital for blistering disease. For all patients, peripheral blood samples were collected by venipuncture in an EDTA-coated vacutainer tube (BD Biosciences, San Jose, CA, USA) and kept in ice until plasma separation (within 30 min). Blood was centrifuged at 1,000 × *g* for 10 min and plasma was aliquoted (0.5 ml per tube) and stored at 80°C until analysis. Among them, 9 did not receive treatment by the time they were diagnosed and samples were collected (untreated subjects), 45 were under treatment with immunosuppressant drugs (treated subjects) (Table S1 in Supplementary Material), and 10 remissive subjects were treated at least 1 year before samples were collected, did not display any features of disease activity nor were taking any further medication (post-treatment subjects). Treated subjects (*n* = 45) were further subcategorized in three groups (group I—29, II—6, and III—10 patients) according to the level of cutaneous lesions. The extension of cutaneous damage was assessed according to the Wallace’s rule of nines (15). 39 subjects, age-matched, originated from the same areas without diagnosis of autoimmune, inflammatory or infectious diseases, neoplastic disorders, or allergies, composed the control group. Furthermore, none of the individuals from the control group had any familial case of pemphigus. Patients diagnosed with FS had clinical, pathological, and/or serological diagnosis. Informed consent was obtained for experimentation with all human subjects. The median age of FS patients in our study was 36 years old with no predominance of sex. The majority of patients were white or mulattoes (76.6%). The number of patients from urban area (40.6%) or from urban area with frequent contact with rural areas (51.6%) was higher than those living in rural area (7.8%) (Table 1).

**Table 1:** Gender, skin color, and housing area of fogo selvagem patients and their control counterparts.

	Control	PF patients	*p* Value
Age (±SD years)	37.85 ± 13.75	37.73 ± 18.51	0.97^a^
Gender	17M/22F	32M/32F	0.2^b^
**Skin color**			
Mulattoes (*n*)	10	20	
White (*n*)	23	29	0.38^c^
Black (*n*)	6	15	
**Housing area**			
Urban area (*n*)	18	26	
Urban and rural areas (*n*)	15	33	0.55^c^
Rural area (*n*)	4	5	

*^a^Unpaired t test*.

*^b^Fisher’s exact test*.

*^c^Chi-square test*.

*n, number of individuals in each condition; PF, pemphigus foliaceus; M, male; F, female*.

All procedures were conducted in accordance with the Declaration of Helsinki, with full patient compliance and approved human practices as defined by the Ethics Committee for Human Experimentation of the Federal University of Triângulo Mineiro (UFTM), Uberaba, Minas Gerais, Brazil, protocol number 1.311.730.

### Chemokine and Cytokine Production

The production of IL-1β, IL-5, IL-12, IL-13, IL-15, IL-22, IL-23, IL-33, CCL-10 (IP-10), CCL-2 (MCP-1), and CCL-11 (Eotaxin-1) was assessed in plasma using the enzyme-linked immunosorbent assay (R&D Systems^®^, San Diego, CA, USA), following the manufacturer’s instructions. Cytometric Bead Array (BD Biosciences, San Jose, CA, USA) was used for simultaneous detection of TNF-α, IFN-γ, TGF-β, IL-2, IL-4, IL-6, IL-9, IL-10, IL-17A, CCL-5 (RANTES), CXCL-8 (IL-8), and CCL3 (MIP-1α).

### Data Analysis and Statistics

For all variables, normal distribution and homogeneous variance were tested. The *D’Agostino-Pearson* test was used to assess normality for all variables. In cases of non-Gaussian distribution of data, the non-parametric Mann–Whitney test was applied. Multiple comparisons relating to the medians of values for more than two groups were made using the Kruskal–Wallis non-parametric test followed by Dunn’s test. For correlations the Spearman test was used. The observed differences were considered significant when *p* < 0.05 (5%). Statistical analysis was performed using the Graphpad Prism software (GraphPad Software 6.0, La Jolla, CA, USA).

## Results

### Cytokine and Chemokine Profile in Treated and Non-Treated Subjects

**Figure 2:**
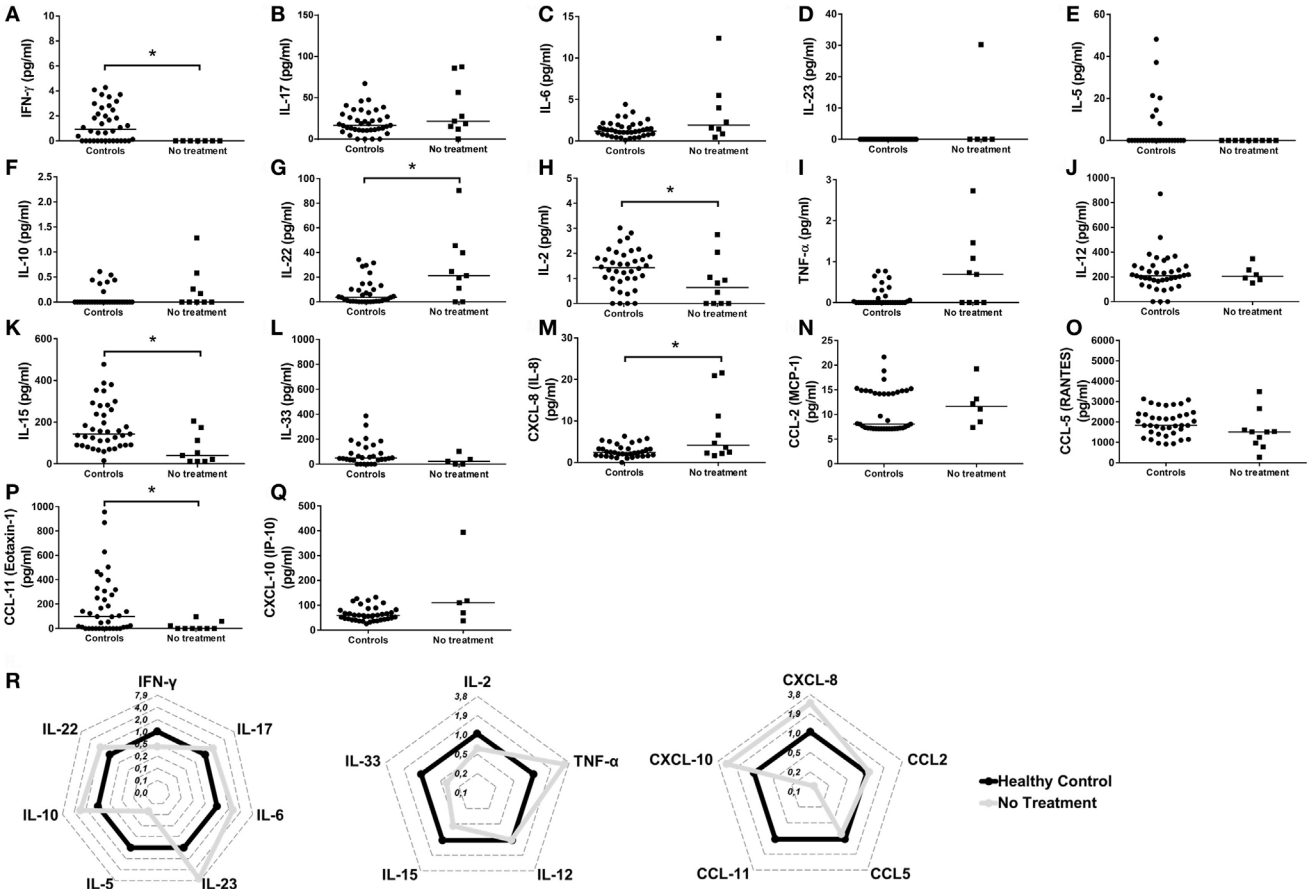
Serum profile of cytokines and chemokines in the pathogenesis of the endemic form (fogo selvagem) of pemphigus foliaceus. Levels of **(A)** IFN-γ, **(B)** IL-17, **(C)** IL-6, **(D)** IL-23, **(E)** IL-5, **(F)** IL-10, **(G)** IL-22, **(H)** IL-2, **(I)** TNF-α, **(J)** IL-12, **(K)** IL-15, **(L)** IL-33, **(M)** CXCL-8/IL-8, **(N)** CCL-2/MCP-1, **(O)** CCL-5/RANTES, **(P)** CCL-11/eotaxin-1, and **(Q)** CXCL-10/IP-10 from healthy donors (controls) and untreated patients with the endemic form (fogo selvagem) of pemphigus foliaceus (no treatment). Lines represent median. *p < 0.05, Mann–Whitney test. **(R)** Radar plot representation of serum cytokine and chemokine profile. Lines highlight the fold change in cytokine levels in pemphigus foliaceus patients (gray line) in relation to healthy donors (black line). Data were obtained by calculating the ratio between the median concentration of each cytokine in the pemphigus foliaceus (fogo selvagem) group and in the healthy group.

Because of the importance of cytokines and chemokines on different physiological and pathological aspects, and due to the lack of a more consistent data concerning the production of these molecules in FS patients, the level of cytokines and chemokines was assessed in untreated FS patients and their healthy control counterparts. Thus, FS patients had lower levels of IFN-γ (Figures 2A,R), IL-2 (Figure 2H), IL-15 (Figure 2K), and CCL-11 (Figures 2P,R) when compared to their control counterparts. On the other hand, the levels of IL-22 (Figure 2G) and CXCL-8/IL-8 (Figures 2M,R) were significantly elevated in FS patients. No differences were detected for the levels of IL-17 (Figure 2B), IL-6 (Figure 2C), IL-23 (Figure 2D), IL-5 (Figure 2E), IL-10 (Figure 2F), TNF-α (Figure 2I), IL-12 (Figure 2J), IL-33 (Figure 2L), CCL-2 (Figure 2N), CCL-5 (Figure 2O), and CXCL-10 (Figure 2Q). The following cytokines/chemokines were not detected: IL-13, TGF-β, IL-4, IL-9, and CCL3 (data not shown). These results suggest a complex interplay between cytokines and chemokines in the pathogenesis of FS.

### The Relationship between Cytokines, Chemokines, and Disease Severity

**Figure 3:**
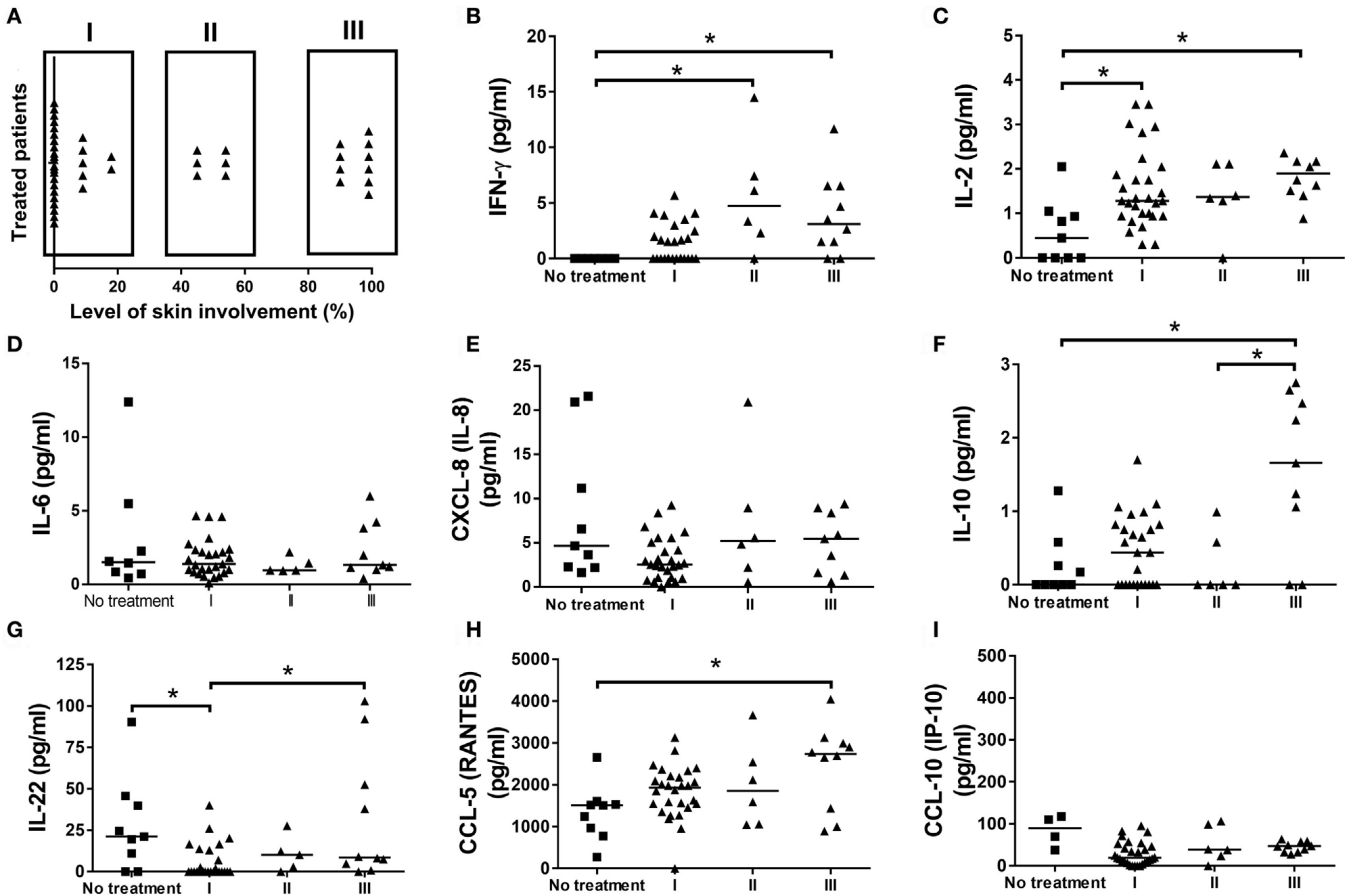
The profile of cytokines, chemokines, and disease severity in patients with the endemic form of pemphigus foliaceus. **(A)** Stratification of fogo selvagem (FS) patients according to the extension of skin lesions. Levels of **(B)** IFN-γ, **(C)** IL-2, **(D)** IL-6, **(E)** IL-8, **(F)** IL-10, **(G)** IL-22, **(H)** CCL-5/RANTES, and **(I)** CCL-10/IP-10, from patients with the endemic form (FS) of pemphigus foliaceus without treatment (No treatment) compared to those taking immunosuppressive therapy with different degrees of lesions in skin (I-III). Lines represent median. *p < 0.05, Kruskal–Wallis test followed by Dunn’s test.

To check for a relationship between disease severity and the levels of cytokines and chemokines, FS patients were stratified according to the extension of lesion (Figure 3A). The greater severity of disease, which means patients with more than 90% of compromised skin (group III), was associated with higher levels of IFN-γ (Figure 3B), IL-2 (Figure 3C), and CCL-5 (Figure 3H), when compared to the untreated patients. Furthermore, higher levels of IL-22 (Figure 3G) were also verified in group III, when compared to group I, and in untreated patients compared to group I. The greater severity of disease was also related to higher levels of IL-10 when compared to group II and untreated patients (Figure 3F). No differences were registered for the levels of IL-6 (Figure 3D), CXCL-8/IL-8 (Figure 3E), and CCL-10/IP-10 (Figure 3I). The following cytokines/chemokines were not detected: TGF-β, IL-4, IL-9, IL-23, IL-13, and CCL3 (data not shown). These results pointed to an influence of severity of disease on cytokine and chemokine production in FS patients.

### Cytokine and Chemokine Interplay after Treatment with Immunosuppressant Drugs

**Figure 4:**
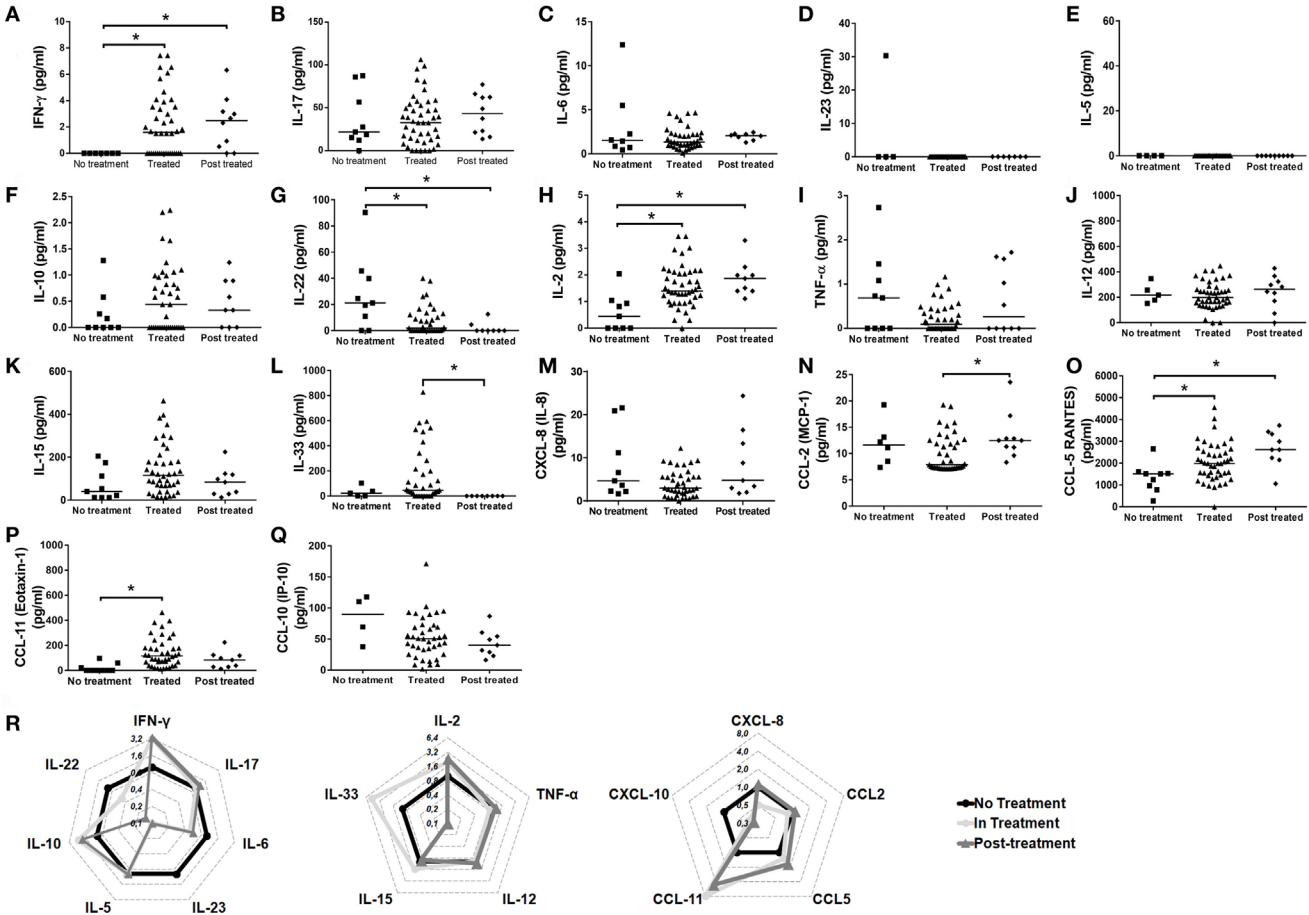
Cytokine and chemokine interplay after treatment with immunosuppressant drugs. Levels of **(A)** IFN-γ, **(B)** IL-17, **(C)** IL-6, **(D)** IL-23, **(E)** IL-5, **(F)** IL-10, **(G)** IL-22, **(H)** IL-2, **(I)** TNF-α, **(J)** IL-12, **(K)** IL-15, **(L)** IL-33, **(M)** CXCL-8/IL-8, **(N)** CCL-2/MCP-1, **(O)** CCL-5/RANTES, **(P)** CCL-11/eotaxin-1, and **(Q)** CXCL-10/IP-10 from patients with the endemic form (fogo selvagem—FS) of pemphigus foliaceus under different conditions of treatment (no treatment, treated, and post-treatment). Lines represent median. *p < 0.05, Kruskal–Wallis test followed by Dunn’s test. **(R)** Radar plot representation of serum cytokine and chemokine profile of not treated (black lines), in treatment (light gray) and post-treatment (dark gray) FS patients. Data were obtained by calculating the ratio between the median concentrations of each cytokine in the pemphigus foliaceus (FS) patients under- or post-treatment versus the untreated group.

Next, to verify if immunosuppressant therapy may induce long-lasting effects on cytokine and chemokine levels, we evaluated the cytokine and chemokine levels in remissive patients and compared it with FS subjects before and during treatment with immunosuppressive therapy. Treatment induced and maintained the production of IFN-γ and IL-2 (Figures 4A,H, respectively). However, though the production of IL-33 (Figures 4L,R) and CCL-11 (Figures 4P,R) was induced by treatment, the higher levels of these molecules were not kept after withdrawal of therapy. The levels of the chemokine CCL-5 (Figure 4O) were higher in FS patients after treatment. The absence of treatment in FS patients was especially associated with higher levels of IL-22 (Figures 4G,R). No differences were observed between the conditions of treatment for the production of IL-17 (Figure 4B), IL-6 (Figure 4C), IL-23 (Figure 4D), IL-5 (Figure 4E), IL-10 (Figure 4F), TNF-α (Figure 4I), IL-12 (Figure 4J), IL-15 (Figure 4K), CXCL-8 (Figure 4M), CCL-2 (Figure 4N), and CCL-10/IP-10 (Figures 4Q,R). The following cytokines/chemokines were not detected: IL-13, TGF-β, IL-4, IL-9, and CCL3 (data not shown). These results suggest that immunosuppressant therapy may induce long-lasting effects on the production of cytokines and chemokines.

## Discussion

In recent years, some efforts have been made to elucidate the role of cytokines and chemokines in the pathogenesis of FS; however, the findings described so far are limited, and their implications in disease outcome were not fully elucidated yet. In our study, the plasma levels of over 17 cytokines and 6 chemokines were evaluated in FS patients. The results pointed to a putative role of IL-22 in the pathogenesis of FS, and to a supposed protective function of especially Th1-related cytokines and Th2 chemokines in serum.

The most affected regions in Brazil include the mid-western and the southeastern states, including Minas Gerais (3, 16), where the majority of patients analyzed in our study came from. Furthermore, FS has been primarily described as predominant over people from rural areas, being uncommonly described in urban areas (3). Since its first description, the etiology of FS has been mainly attributed to environmental triggers present in rural areas, which can be different based on geographical distribution and genetic inheritance (17, 18). Despite the description of FS as primarily found in rural areas, our study suggested an urbanization of this disease. This scenario could be at least partly attributed to the presence of strong environmental triggers in urban areas, including stress, lifestyle, and pollution, which were already described as potential triggers for other autoimmune diseases such as those affecting thyroid (19), systemic lupus erythematosus, and type 1 diabetes (20), for example. Additionally, the saliva of sand flies contains some components like LJM11, which is a salivary protein from the sand fly *Lutzomyia longipalpis* (21), able to cross-react to anti-Dsg1 monoclonal autoantibodies from FS patients. This phenomenon, in turn, induced a production of autoantibodies targeting Dsg1 in mice previously immunized with the salivary protein (1, 21, 22). Accordingly, the so-called “urbanization” of FS observed in our study, can be also attributed to changes in the behavior of the insects moving from sylvatic areas to urban areas as a consequence of social–environmental alterations (23, 24). Therefore, it is possible to consider the role of insect-derived saliva proteins, like LJM11, in the onset of a pathogenic response, leading to FS.

The complex interplay between cytokines and chemokines that dictates disease outcome in FS is still a matter of investigation. In our study, the comparison between healthy subjects and FS patients suggested a role of IL-22, and to a less extent IL-8, in the FS. Healthy subjects had higher levels of IFN-γ and IL-2. Similarly, elevated levels of IFN-γ and IL-2, along with a reduction in IL-22, were observed in FS patients under treatment or after treatment withdrawn, when compared to untreated patients. A different study, also pointed to a reduction in serum of IL-2 and IFN-γ in FS patients when compared to their healthy control counterparts (9). Though in a different pemphigus entity, PV-treated patients also showed elevated levels of IFN-γ (25). Additionally, the inverse relationship between IL-22 and IFN-γ was described in patients with active psoriasis (26). IL-22 is especially produced by Th17 and Th22 cells (27) and is involved in several processes including the regulation of tissue cells in skin, kidney, pancreas, small intestine, liver, colon, and respiratory system (26). Despite the beneficial role of IL-22 in the aforementioned tissues, this cytokine, depending on the context, may present a pro-inflammatory role. For this reason, it has been implicated in the pathogenesis of different inflammatory skin disorders including atopic dermatitis (28), psoriasis (29) and systemic lupus erythematosus with skin manifestations (30). However, the participation of IL-22 in the pathogenesis of FS is still not elucidated and needs to be further explored. In skin, despite the absence of effect over skin fibroblasts, endothelial cells, and melanocytes, proliferation of keratinocytes, epidermal hyperplasia, and abrogated keratinocyte terminal differentiation are mediated by IL-22, mainly derived from Th22 cells (31). IL-22 has shown *in vitro*, great ability to downregulate key genes in keratinocyte differentiation, including loricrin, filaggrin, and involucrin, which resulted in striking acanthosis (32, 33). Because of the effect of IL-22 in skin, higher levels in both skin and serum have been related to disease worsening in patients with active psoriasis (34, 35). Even though psoriasis and PF are considered to be distinct entities, in some cases, they can occur together in the same patient. In these cases, PF has been developed in untreated or chronic psoriatic patients (36–42). However, we cannot exclude the participation of genetics in this context, the presence of HLA DRB1 alleles, which have been described to be related with both psoriasis (43) and PF/FS (44, 45), could possibly explain the concomitant occurrence of both diseases. Although we have not determined the presence of the HLA DRB1 alleles in our study, the importance of IL-22 in the pathogenesis of FS cannot be underestimated and needs to be further explored. Additionally, we cannot underestimate the importance of the balance between IL-22, IL-2, and IFN-γ, neither the role of IL-22, on disease onset and progression. As we have pointed out here, in treated patients (even after treatment withdrawn), the opposite relationship between the higher levels of IL-2 and IFN-γ and lower levels of IL-22, appeared to be related to disease amelioration. We believe that in FS, IL-2 and IFN-γ may exert a protective role antagonizing the effects of IL-22.

Despite the crucial role of cytokines in the pathogenesis of FS, chemokines are also important players in this scenario. Our results pointed to higher levels of CXCL-8/IL-8 in FS patients. IL-8 is a chemokine produced during inflammatory stimulation, and its main function is to chemoatract and activate neutrophils (46). Though this chemokine is widely studied owing to its role in neutrophil recruitment and inflammation, data concerning the participation of IL-8 in the pathogenesis of FS still needs to be clarified. In a rare subset of pemphigus, known as pemphigus herpetiformis (PH), in which IgG autoantibodies targeting Dsg1 seems to be related to the development of disease, it was suggested that these autoantibodies were able to induce overexpression of IL-8 in keratinocytes (47). Cultured keratinocytes from these patients showed increased production and secretion of cytoplasmic IL-8 after stimulation with autoantibodies derived from PH patients (47), which reinforces the effect of autoantibodies targeting Dsg1 on the production of IL-8. Similarly, the blister formation in bullous pemphigoid was associated with higher levels in serum of IL-6 and IL-8 (48, 49). In contrast, in other subsets of pemphigus, pemphigus vulgaris, higher levels of IL-8 were observed in PV-treated patients (25). Although we have not investigated the relationship between the effects of autoantibodies targeting Dsg1 on the production of IL-8 in our study, we cannot exclude this possibility. Moreover, though not as important as IL-22, IL-8 seems also to be related to disease activity, and together with the former, may represent a potential target for new therapeutic approaches. However, such role of IL-22 and IL-8 in FS pathogenesis needs to be further clarified.

In our study, CCL-5/RANTES was induced and maintained by immunosuppressant drugs even after treatment withdrawn. However, its role in the development of FS is not fully explored. CCL-5 is a chemokine with strong capacity to recruit monocytes, T cells, and eosinophils, acting via the receptors CCR1, CCR3, and CCR5 (50). Its expression in other forms of pemphigus is controversial. Patients diagnosed with bullous pemphigoid, pemphigus, and dermatitis herpetiformis showed no differences for the presence of IL-8 and RANTES in the skin lesions (51) or in serum (52), when compared to their control counterparts. Interestingly, the higher levels of CCL-5, IFN-γ, and IL-2 found in treated patients in our study were followed by a reduction in IL-22. In patients with lung inflammation, higher levels of IL-22 were shown to be able to suppress IFN-γ-induced secretion of CCL-5 (53). The effects of immunosuppressant drugs like prednisone, one of the most prescribed therapies to treat FS, were shown to be involved in the reduction of IL-22 in patients with systemic lupus erythematosus (54). Therefore, we believe that immunosuppressive therapy influenced this balance, reducing the levels of IL-22, and thus favoring the production of IFN-γ-derived CCL-5. Nevertheless, the mechanisms behind this modulation were not explored in our study. Additionally, it is difficult to address the consistencies/inconsistencies between different subtypes of pemphigus. This is probably due to the distinctions in etiology, physiopathology, geographical area, incidence, and prevalence observed in each case.

We believe that our study is the most detailed and complete study on the effects of cytokines and chemokines in the pathogenesis of the endemic form (FS) of PF. Our results pointed to a putative role of IL-22 in the pathogenesis of FS, and to the importance of immunosuppressive therapy to reestablish the immune balance in FS patients. Ultimately, we believe that this study may provide a better knowledge of the cytokines and chemokines involved in disease outcome, besides suggesting IL-22 as a hypothetical target to be explored in future studies. Moreover, our study demonstrates, for the first time, the urbanization of the disease. These data are also important because future preventive decisions must take into account the environmental triggers in urban areas that may be related with the onset of the disease.

## Ethics Statement

This study was carried out in strict accordance with the principles and guidelines adopted by the Declaration of Helsinki, with full patient compliance and approved human practices as defined by the Ethics Committee for Human Experimentation of the Federal University of Triângulo Mineiro (UFTM), Uberaba, Minas Gerais, Brazil, protocol number 1.300.898.

## Author Contributions

RT, MS, FA, VJR, AR, HS-C, and CO conceived and designed the experiments; RT, HS-C, and CO wrote the paper with the assistance of all the authors. RT, MS, DS, JC, and HS-C performed the experiments. RT, MS, DS, JC, FA, VJR, AR, HS-C, and CO analyzed the data. RT, MS, DS, JC, FA, VJR, AR, HS-C, and CO provided critical review for important intellectual content. The final version of the manuscript was approved by all authors.

## Conflict of Interest Statement

The authors declare that the research was conducted in the absence of any commercial or financial relationships that could be construed as a potential conflict of interest.

## References

[1] Aoki V Rivitti EA Diaz LA Cooperative Group on Fogo Selvagem Research. Update on fogo selvagem, an endemic form of pemphigus foliaceus. J Dermatol (2015) 42:18–26. 10.1111/1346-8138.12675 25558948 PMC4496802

[2] Alpsoy E Akman-Karakas A Uzun S. Geographic variations in epidemiology of two autoimmune bullous diseases: pemphigus and bullous pemphigoid. Arch Dermatol Res (2015) 307:291–8. 10.1007/s00403-014-1531-1 25589418

[3] Diaz LA Sampaio SA Rivitti EA Martins CR Cunha PR Lombardi C Endemic pemphigus foliaceus (fogo selvagem): II. Current and historic epidemiologic studies. J Invest Dermatol (1989) 92:4–12. 10.1111/1523-1747.ep13070394 2642512

[4] Silvestre MC Netto JCA Endemic pemphigus foliaceus: social and demographical characteristics and incidence in the microregions of Goias, based on patients seen at the Tropical Diseases Hospital, Goiania-Goias. An Bras Dermatol (2005) 80:261–6. 10.1590/S0365-05962005000300006

[5] Oliveira LA Marquart-Filho A Trevilato G Timoteo RP Mukai M Roselino AM Anti-desmoglein 1 and 3 autoantibody levels in endemic pemphigus foliaceus and pemphigus vulgaris from Brazil. Clin Lab (2016) 62:1209–16. 10.7754/Clin.Lab.2015.150628 28164651

[6] Ludwig RJ Vanhoorelbeke K Leypoldt F Kaya Z Bieber K McLachlan SM Mechanisms of autoantibody-induced pathology. Front Immunol (2017) 8:603. 10.3389/fimmu.2017.00603 28620373 PMC5449453

[7] Satyam A Khandpur S Sharma VK Sharma A. Involvement of T(H)1/T(H)2 cytokines in the pathogenesis of autoimmune skin disease-pemphigus vulgaris. Immunol Invest (2009) 38:498–509. 10.1080/08820130902943097 19811408

[8] Chriguer RS Roselino AM de Castro M. Glucocorticoid sensitivity and proinflammatory cytokines pattern in pemphigus. J Clin Immunol (2012) 32:786–93. 10.1007/s10875-012-9679-y 22407150

[9] Zeoti DM Figueiredo JF Chiossi MP Roselino AM. Serum cytokines in patients with Brazilian pemphigus foliaceus (fogo selvagem). Braz J Med Biol Res (2000) 33:1065–8. 10.1590/S0100-879X2000000900012 10973139

[10] Rocha-Rodrigues DB Paschoini G Pereira SA dos Reis MA Teixeira Vde P Rodrigues Junior V. High levels of interleukin-1 in patients with endemic pemphigus foliaceus. Clin Diagn Lab Immunol (2003) 10:741–3. 10.1128/CDLI.10.5.741-743.2003 12965897 PMC193914

[11] Singh TP Schon MP Wallbrecht K Gruber-Wackernagel A Wang XJ Wolf P. Involvement of IL-9 in Th17-associated inflammation and angiogenesis of psoriasis. PLoS One (2013) 8:e51752. 10.1371/journal.pone.0051752 23335955 PMC3546056

[12] Mabuchi T Takekoshi T Hwang ST Epidermal CCR6+ gammadelta T cells are major producers of IL-22 and IL-17 in a murine model of psoriasiform dermatitis. J Immunol (2011) 187:5026–31. 10.4049/jimmunol.1101817 21984702

[13] Grattan C Evidence of an association between bullous pemphigoid and psoriasis. Br J Dermatol (1985) 113:281–3. 10.1111/j.1365-2133.1985.tb02079.x 3904804

[14] Tsai TF Wang TS Hung ST Tsai PI Schenkel B Zhang M Epidemiology and comorbidities of psoriasis patients in a national database in Taiwan. J Dermatol Sci (2011) 63:40–6. 10.1016/j.jdermsci.2011.03.002 21543188

[15] Hettiaratchy S Papini R Initial management of a major burn: II – Assessment and resuscitation. BMJ (2004) 329:101–3. 10.1136/bmj.329.7457.101 15242917 PMC449823

[16] Hans-Filho G dos Santos V Katayama JH Aoki V Rivitti EA Sampaio SA An active focus of high prevalence of fogo selvagem on an Amerindian reservation in Brazil. Cooperative Group on Fogo Selvagem Research. J Invest Dermatol (1996) 107:68–75. 10.1111/1523-1747.ep12298213 8752842

[17] Empinotti JC Aoki V Filgueira A Sampaio SA Rivitti EA Sanches JA Jr Clinical and serological follow-up studies of endemic pemphigus foliaceus (fogo selvagem) in Western Parana, Brazil (2001–2002). Br J Dermatol (2006) 155:446–50. 10.1111/j.1365-2133.2006.07302.x 16882187

[18] Sampaio SA Rivitti EA Aoki V Diaz LA. Brazilian pemphigus foliaceus, endemic pemphigus foliaceus, or fogo selvagem (wild fire). Dermatol Clin (1994) 12:765–76. 7805306

[19] Brent GA. Environmental exposures and autoimmune thyroid disease. Thyroid (2010) 20:755–61. 10.1089/thy.2010.1636 20578899 PMC2935336

[20] Schmidt CW Questions persist: environmental factors in autoimmune disease. Environ Health Perspect (2011) 119:A249–53. 10.1289/ehp.119-a248 21628113 PMC3114837

[21] Qian Y Jeong JS Maldonado M Valenzuela JG Gomes R Teixeira C Cutting edge: Brazilian pemphigus foliaceus anti-desmoglein 1 autoantibodies cross-react with sand fly salivary LJM11 antigen. J Immunol (2012) 189:1535–9. 10.4049/jimmunol.1200842 22798673 PMC3411885

[22] Qian Y Jeong JS Abdeladhim M Valenzuela JG Aoki V Hans-Filhio G IgE anti-LJM11 sand fly salivary antigen may herald the onset of fogo selvagem in endemic Brazilian regions. J Invest Dermatol (2015) 135:913–5. 10.1038/jid.2014.430 25285921 PMC4323842

[23] Desjeux P. The increase in risk factors for leishmaniasis worldwide. Trans R Soc Trop Med Hyg (2001) 95:239–43. 10.1016/S0035-9203(01)90223-8 11490989

[24] Harhay MO Olliaro PL Costa DL Costa CH. Urban parasitology: visceral leishmaniasis in Brazil. Trends Parasitol (2011) 27:403–9. 10.1016/j.pt.2011.04.001 21596622

[25] Timoteo RP da Silva MV Miguel CB Silva DA Catarino JD Rodrigues Junior V Th1/Th17-related cytokines and chemokines and their implications in the pathogenesis of pemphigus vulgaris. Mediators Inflamm (2017) 2017:7151285. 10.1155/2017/7151285 28321152 PMC5340942

[26] Wolk K Kunz S Witte E Friedrich M Asadullah K Sabat R. IL-22 increases the innate immunity of tissues. Immunity (2004) 21:241–54. 10.1016/j.immuni.2004.07.007 15308104

[27] Trifari S Kaplan CD Tran EH Crellin NK Spits H Identification of a human helper T cell population that has abundant production of interleukin 22 and is distinct from T(H)-17, T(H)1 and T(H)2 cells. Nat Immunol (2009) 10:864–71. 10.1038/ni.1770 19578368

[28] Nograles KE Zaba LC Shemer A Fuentes-Duculan J Cardinale I Kikuchi T IL-22-producing “T22” T cells account for upregulated IL-22 in atopic dermatitis despite reduced IL-17-producing TH17 T cells. J Allergy Clin Immunol (2009) 123:1244–52.e2. 10.1016/j.jaci.2009.03.041 19439349 PMC2874584

[29] Benham H Norris P Goodall J Wechalekar MD FitzGerald O Szentpetery A Th17 and Th22 cells in psoriatic arthritis and psoriasis. Arthritis Res Ther (2013) 15:R136. 10.1186/ar4317 24286492 PMC3978433

[30] Yang XY Wang HY Zhao XY Wang LJ Lv QH Wang QQ. Th22, but not Th17 might be a good index to predict the tissue involvement of systemic lupus erythematosus. J Clin Immunol (2013) 33:767–74. 10.1007/s10875-013-9878-1 23435610

[31] Wolk K Haugen HS Xu W Witte E Waggie K Anderson M IL-22 and IL-20 are key mediators of the epidermal alterations in psoriasis while IL-17 and IFN-gamma are not. J Mol Med (Berl) (2009) 87:523–36. 10.1007/s00109-009-0457-0 19330474

[32] Sa SM Valdez PA Wu J Jung K Zhong F Hall L The effects of IL-20 subfamily cytokines on reconstituted human epidermis suggest potential roles in cutaneous innate defense and pathogenic adaptive immunity in psoriasis. J Immunol (2007) 178:2229–40. 10.4049/jimmunol.178.11.7487-a 17277128

[33] Boniface K Bernard FX Garcia M Gurney AL Lecron JC Morel F. IL-22 inhibits epidermal differentiation and induces proinflammatory gene expression and migration of human keratinocytes. J Immunol (2005) 174:3695–702. 10.4049/jimmunol.174.6.3695 15749908

[34] Boniface K Guignouard E Pedretti N Garcia M Delwail A Bernard FX A role for T cell-derived interleukin 22 in psoriatic skin inflammation. Clin Exp Immunol (2007) 150:407–15. 10.1111/j.1365-2249.2007.03511.x 17900301 PMC2219373

[35] Wolk K Witte E Warszawska K Schulze-Tanzil G Witte K Philipp S The Th17 cytokine IL-22 induces IL-20 production in keratinocytes: a novel immunological cascade with potential relevance in psoriasis. Eur J Immunol (2009) 39:3570–81. 10.1002/eji.200939687 19830738

[36] Lee CW Ro YS Kim JH Kim JH Concurrent development of pemphigus foliaceus and psoriasis. Int J Dermatol (1985) 24:316–7. 10.1111/j.1365-4362.1985.tb05792.x 4018981

[37] Tomasini D Cerri A Cozzani E Berti E. Development of pemphigus foliaceus in a patient with psoriasis: a simple coincidence? Eur J Dermatol (1998) 8:56–9. 9649683

[38] Perez GL Agger WA Abellera RM Dahlberg P Pemphigus foliaceus coexisting with IgA nephropathy in a patient with psoriasis vulgaris. Int J Dermatol (1995) 34:794–6. 10.1111/j.1365-4362.1995.tb04400.x 8543414

[39] Yokoo M Oka D Ueki H Coexistence of psoriasis vulgaris and pemphigus foliaceus. Dermatologica (1989) 179:222–3. 10.1159/000248369 2620759

[40] Giomi B Cardinali C Pestelli E Caproni M Fabbri P Pemphigus foliaceus developing on pre-existing psoriasis: a supposed pathogenetic linkage. Acta Derm Venereol (2004) 84:82–3. 10.1080/00015550310020567 15040491

[41] Lee CW Ro YS. Pemphigus developed on preexisting dermatoses. J Dermatol (1994) 21:213–5. 10.1111/j.1346-8138.1994.tb01724.x 8014278

[42] Kwon HH Kwon IH Chung JH Youn JI. Pemphigus foliaceus associated with psoriasis during the course of narrow-band UVB therapy: a simple coincidence? Ann Dermatol (2011) 23:S281–4. 10.5021/ad.2011.23.S3.S281 22346257 PMC3276776

[43] Cardoso CB Uthida-Tanaka AM Magalhaes RF Magna LA Kraemer MH. Association between psoriasis vulgaris and MHC-DRB, -DQB genes as a contribution to disease diagnosis. Eur J Dermatol (2005) 15:159–63. 15908298

[44] Pavoni DP Roxo VM Marquart Filho A Petzl-Erler ML. Dissecting the associations of endemic pemphigus foliaceus (fogo selvagem) with HLA-DRB1 alleles and genotypes. Genes Immun (2003) 4:110–6. 10.1038/sj.gene.6363939 12618858

[45] Brochado MJ Nascimento DF Campos W Deghaide NH Donadi EA Roselino AM Differential HLA class I and class II associations in pemphigus foliaceus and pemphigus vulgaris patients from a prevalent Southeastern Brazilian region. J Autoimmun (2016) 72:19–24. 10.1016/j.jaut.2016.04.007 27178774

[46] Xie K. Interleukin-8 and human cancer biology. Cytokine Growth Factor Rev (2001) 12:375–91. 10.1016/S1359-6101(01)00016-8 11544106

[47] O’Toole EA Mak LL Guitart J Woodley DT Hashimoto T Amagai M Induction of keratinocyte IL-8 expression and secretion by IgG autoantibodies as a novel mechanism of epidermal neutrophil recruitment in a pemphigus variant. Clin Exp Immunol (2000) 119:217–24. 10.1046/j.1365-2249.2000.01104.x 10606986 PMC1905536

[48] Inaoki M Takehara K. Increased serum levels of interleukin (IL)-5, IL-6 and IL-8 in bullous pemphigoid. J Dermatol Sci (1998) 16:152–7. 10.1016/S0923-1811(97)00044-3 9459128

[49] Schmidt E Reimer S Kruse N Jainta S Brocker EB Marinkovich MP Autoantibodies to BP180 associated with bullous pemphigoid release interleukin-6 and interleukin-8 from cultured human keratinocytes. J Invest Dermatol (2000) 115:842–8. 10.1046/j.1523-1747.2000.00141.x 11069622

[50] Schall TJ Bacon K Toy KJ Goeddel DV. Selective attraction of monocytes and T lymphocytes of the memory phenotype by cytokine RANTES. Nature (1990) 347:669–71. 10.1038/347669a0 1699135

[51] Bornscheuer E Zillikens D Schroder JM Sticherling M. Lack of expression of interleukin 8 and RANTES in autoimmune bullous skin diseases. Dermatology (1999) 198:118–21. 10.1159/000018085 10325455

[52] Nakashima H Fujimoto M Asashima N Watanabe R Kuwano Y Yazawa N Serum chemokine profile in patients with bullous pemphigoid. Br J Dermatol (2007) 156:454–9. 10.1111/j.1365-2133.2006.07601.x 17300233

[53] Pennino D Bhavsar PK Effner R Avitabile S Venn P Quaranta M IL-22 suppresses IFN-gamma-mediated lung inflammation in asthmatic patients. J Allergy Clin Immunol (2013) 131:562–70. 10.1016/j.jaci.2012.09.036 23174657

[54] Lin J Yue LH Chen WQ. Decreased plasma IL-22 levels and correlations with IL-22-producing T helper cells in patients with new-onset systemic lupus erythematosus. Scand J Immunol (2014) 79:131–6. 10.1111/sji.12135 24313261

