# Supplementary material for: Cytokine and Chemokines Alterations in the Endemic Form of Pemphigus Foliaceus (Fogo Selvagem)
Source: Front Immunol. 2017 Aug 14;8:978. doi: 10.3389/fimmu.2017.00978 (PMC5557772; doi:10.3389/fimmu.2017.00978)
Supplement: Supplementary file 1 [file table_1.docx]

**Supplemental material 1 -** Clinical features and treatment of Fogo selvagem patients.

| **Patient** | **Age**  **(range)** | **Level of skin involvement (%)** | **Medication**  **(mg/day)** |
| --- | --- | --- | --- |
| 1 | 75-80 | 0 | Untreated |
| 2 | 50-55 | 0 | Untreated |
| 3 | 45-50 | 0 | Untreated |
| 4 | 45-50 | 0 | Untreated |
| 5 | 65-70 | 0 | Untreated |
| 6 | 70-75 | 0 | Untreated |
| 7 | 65-70 | 0 | Untreated |
| 8 | 55-60 | 0 | Untreated |
| 9 | 40-45 | 0 | Untreated |
| 10 | 30-35 | 0 | Untreated |
| 11 | 10-15 | 0 | Prednisone (20mg),  Diaminodiphenyl sulfone(100mg) |
| 12 | 45-50 | 9 | Prednisone (5mg) |
| 13 | 10-15 | 0 | Prednisone (5mg),  Vitamin D (20.000 units) |
| 14 | 15-20 | 9 | Prednisone (5mg) |
| 15 | 75-80 | 9 | Prednisone (10mg) |
| 16 | 50-55 | 9 | Deflazacort (6mg),  Diaminodiphenyl sulfone (50mg) |
| 17 | 25-30 | 0 | Prednisone (5mg) |
| 18 | 30-35 | 9 | Prednisone (5mg) |
| 19 | 20-25 | 0 | Prednisone (20mg),  Vitamin D (20.000 units) |
| 20 | 45-50 | 0 | Prednisone (30mg),  Diaminodiphenyl sulfone (100mg),  Vitamin D (20.000 units) |
| 21 | 65-70 | 0 | Prednisone (10mg),  Diaminodiphenyl sulfone (100mg),  Vitamin D (20.000 units) |
| 22 | 45-50 | 0 | Prednisone (60 mg) |
| 23 | 30-35 | 45 | Prednisone (20mg),  Diaminodiphenyl sulfone (100mg) |
| 24 | 30-35 | 0 | Prednisone (10mg),  Diaminodiphenyl sulfone (50mg) |
| 25 | 10-15 | 0 | Prednisone (20mg),  Betamethasone Dipropionate/Gentamicin Sulfate (topical application) |
| 26 | 15-20 | 0 | Prednisone (20mg) |
| 27 | 25-30 | 0 | Daflazacort (30mg),  Diaminodiphenyl sulfone (150mg) |
| 28 | 10-15 | 0 | Prednisone (10mg) |
| 29 | 45-50 | 0 | Prednisone (10mg) |
| 30 | 30-35 | 0 | Prednisone (60mg),  Diaminodiphenyl sulfone (50mg) |
| 31 | 35-40 | 0 | Prednisone (60mg),  Diaminodiphenyl sulfone (50mg) |
| 32 | 30-35 | 0 | Prednisone (60mg),  Diaminodiphenyl sulfone (50mg) |
| 33 | 15-20 | 18 | Diaminodiphenyl sulfone (100mg),  Ranitidine (dosage not show),  Vitamin D (20.000 units) |
| 34 | 35-40 | 45 | Diaminodiphenyl sulfone (100mg) |
| 35 | 30-35 | 54 | Prednisone (60mg),  Diaminodiphenyl sulfone (100mg),  Vitamin D (20.000 units) |
| 36 | 40-45 | 0 | Diaminodiphenyl sulfone (50mg),  Betamethasone 17α,21-dipropionate and 21-disodium phosphate |
| 37 | 45-50 | 0 | Prednisone (15mg) |
| 38 | 45-50 | 54 | Prednisone (20mg),  Diaminodiphenyl sulfone (100mg) |
| 39 | 55-60 | 45 | Prednisone (50mg),  Levothyroxine sodium,  Hydroxychloroquine (400mg) |
| 40 | 25-30 | 0 | Prednisone (10mg) |
| 41 | 35-40 | 90 | Prednisone (10mg) |
| 42 | 55-60 | 18 | Prednisone (40mg) |
| 43 | 35-40 | 0 | Prednisone (40mg) |
| 44 | 30-35 | 0 | Prednisone (20mg),  Azatioprine (100mg) |
| 45 | 15-20 | 0 | Prednisone (20mg),  Hydroxychloroquine (250mg) |
| 46 | 35-40 | 90 | Prednisone (60mg),  Diaminodiphenyl sulfone (100mg) |
| 47 | 45-50 | 99 | Prednisone (60mg),  Diaminodiphenyl sulfone (100mg),  Vitamin D (20.000 units) |
| 48 | 35-40 | 90 | Prednisone (80mg),  Diaminodiphenyl sulfone (50mg) |
| 49 | 15-20 | 54 | Dalfazacort (15mg),  Diaminodiphenyl sulfone (150mg) |
| 50 | 10-15 | 99 | Prednisone (40mg),  Diaminodiphenyl sulfone (50mg),  Ranitidine (150mg) |
| 51 | 15-20 | 99 | Prednisone (20mg),  Diaminodiphenyl sulfone (100mg) |
| 52 | 55-60 | 99 | Prednisone (20mg),  Diaminodiphenyl sulfone (100mg) |
| 53 | 30-35 | 90 | Prednisone (50mg),  Diaminodiphenyl sulfone(100mg) |
| 54 | 15-20 | 99 | Prednisone (60mg), |
| 55 | 15-20 | 99 | Prednisone (60mg),  Naproxen (500mg) |
| 56 | 15-20 | 9 | Untreated |
| 57 | 55-60 | 9 | Untreated |
| 58 | 15-20 | 9 | Untreated |
| 59 | 35-40 | 9 | Untreated |
| 60 | 30-35 | 18 | Untreated |
| 61 | 80-85 | Undetermined | Untreated |
| 62 | 20-25 | Undetermined | Untreated |
| 63 | 55-60 | Undetermined | Untreated |
| 64 | 20-25 | Undetermined | Untreated |

Serum from 64 patients diagnosed with Fogo Selvagem was used in this study. Among them, 9 did not receive treatment by the time they were diagnosed and samples were collected (untreated subjects); 45 were under treatment with immunosuppressant drugs (treated subjects), and 10 remissive subjects were treated at least one year before samples were collected, did not display any features of disease activity nor were taking any further medication (post-treatment subjects).
